# Ovarian cancer symptom awareness and anticipated delayed presentation in a population sample

**DOI:** 10.1186/1471-2407-14-171

**Published:** 2014-03-10

**Authors:** Kate E Brain, Stephanie Smits, Alice E Simon, Lindsay J Forbes, Chris Roberts, Iain J Robbé, John Steward, Ceri White, Richard D Neal, Jane Hanson

**Affiliations:** 1 Cochrane Institute of Primary Care and Public Health, Neuadd Meirionydd, School of Medicine, Cardiff University, Heath Park, Cardiff CF14 4YS, UK; 2 School of Health Sciences, City University London, London, UK; 3 CR-UK Health Behaviour Research Centre, University College London, London, UK; 4 Promoting Early Presentation Group, King’s College London, London, UK; 5 Knowledge and Analytical Services, Welsh Government, Cardiff, UK; 6 Faculty of Medicine, Memorial University, Newfoundland, Canada; 7 Welsh Cancer Intelligence and Surveillance Unit, Cardiff, UK; 8 North Wales Centre for Primary Care Research, Bangor University, Bangor, UK; 9 Cancer National Specialist Advisory Group, Cardiff, UK

**Keywords:** Ovarian cancer, Symptoms, Awareness, Anticipated delay

## Abstract

**Background:**

While ovarian cancer is recognised as having identifiable early symptoms, understanding of the key determinants of symptom awareness and early presentation is limited. A population-based survey of ovarian cancer awareness and anticipated delayed presentation with symptoms was conducted as part of the International Cancer Benchmarking Partnership (ICBP).

**Methods:**

Women aged over 50 years were recruited using random probability sampling (n = 1043). Computer-assisted telephone interviews were used to administer measures including ovarian cancer symptom recognition, anticipated time to presentation with ovarian symptoms, health beliefs (perceived risk, perceived benefits/barriers to early presentation, confidence in symptom detection, ovarian cancer worry), and demographic variables. Logistic regression analysis was used to identify the contribution of independent variables to anticipated presentation (categorised as < 3 weeks or ≥ 3 weeks).

**Results:**

The most well-recognised symptoms of ovarian cancer were post-menopausal bleeding (87.4%), and persistent pelvic (79.0%) and abdominal (85.0%) pain. Symptoms associated with eating difficulties and changes in bladder/bowel habits were recognised by less than half the sample. Lower symptom awareness was significantly associated with older age (p ≤ 0.001), being single (p ≤ 0.001), lower education (p ≤ 0.01), and lack of personal experience of ovarian cancer (p ≤ 0.01). The odds of anticipating a delay in time to presentation of ≥ 3 weeks were significantly increased in women educated to degree level (OR = 2.64, 95% CI 1.61 – 4.33, p ≤ 0.001), women who reported more practical barriers (OR = 1.60, 95% CI 1.34 – 1.91, p ≤ 0.001) and more emotional barriers (OR = 1.21, 95% CI 1.06 – 1.40, p ≤ 0.01), and those less confident in symptom detection (OR = 0.56, 95% CI 0.42 – 0.73, p ≤ 0.001), but not in those who reported lower symptom awareness (OR = 0.99, 95% CI 0.91 – 1.07, p = 0.74).

**Conclusions:**

Many symptoms of ovarian cancer are not well-recognised by women in the general population. Evidence-based interventions are needed not only to improve public awareness but also to overcome the barriers to recognising and acting on ovarian symptoms, if delays in presentation are to be minimised.

## Background

Ovarian cancer accounts for 4% of all cancers diagnosed in women, with over 200,000 new cases each year worldwide [1] and one year survival lowest for women in the UK [2]. Low awareness and negative beliefs about cancer are implicated in delayed presentation of cancer symptoms, leading to advanced stage at diagnosis and a lower chance of survival [3-5]. This may especially be the case for ovarian cancer, a less common cancer with large international variations in survival rates [6].

Ovarian cancer is now recognised as having detectable early symptoms including abdominal distension (bloating, increased abdominal size), pelvic and/or abdominal pain, problems with eating (loss of appetite, feeling full quickly), and frequent urination [7,8]. Symptoms are recognised to be present in both early and late stage ovarian cancer, with better prognosis for disease diagnosed at an earlier stage [9]. However, women with ovarian cancer may not be aware that their symptoms were indicative of ovarian cancer and may misattribute them to irritable bowel syndrome, ageing, stress or other benign causes [9,10]. This knowledge provides the basis for practitioner guidelines [11,12], risk assessment tools [13] and information for the public [14-16] aimed at improving ovarian symptom awareness and earlier presentation. Most patients with ovarian cancer present initially to their general practitioner, with around half having had symptoms for more than one month [17]. No screening programmes exist for ovarian cancer; however, there are initiatives to determine whether screening may be effective, including the US-based Symptom Index in combination with biomarkers [18-20] and the UK ovarian cancer screening study which reports in 2015 [21]. Given the lack of an imminent ovarian screening programme or opportunities in other parts of the diagnostic pathway to expedite diagnoses, evidence is needed regarding the determinants of lower awareness and delay in presentation to inform interventions aimed at improving early detection of ovarian cancer. Studies of risk factors for delayed symptomatic presentation in other cancers have highlighted a range of barriers including older age [22-24], lower socio-economic status [25], misinterpreting the seriousness of symptoms [26,27], and fears about what might be found [3].

The present study carried out as part of the International Cancer Benchmarking Partnership (ICBP) which was established in 2010 to investigate the causes of international variation in cancer outcomes. We sought to identify levels of ovarian symptom awareness and demographic risk factors for lower awareness and anticipated delay in a representative population sample of women over age 50. This age group was selected because most cases of ovarian cancer (> 80%) are diagnosed in the over-50s [28]. In addition, we examined the effects of health beliefs on anticipated delay, including perceived benefits and barriers to symptomatic presentation, confidence in detecting symptoms, and perceived risk of ovarian cancer [29-31]. In order to develop interventions which raise cancer awareness without raising anxiety, it was also considered important to examine the potential influence of cancer worry [32]. It was hypothesised that few perceived benefits, more barriers, low confidence, and low worry would be associated with anticipated delay. Since prospective monitoring of actual symptom presentation would require following up an unfeasibly large sample, we used a hypothetical question (“how long it would take you to go to the doctors with a symptom”) as a proxy measure of delayed presentation.

## Methods

The survey was conducted as a subset of the ICBP survey of awareness and beliefs about cancer in adults aged ≥50 years in six countries [33]. For the present analyses, we used data from female respondents in Wales. Ethical approval was obtained from Cardiff University School of Medicine Research Ethics Committee. The survey was carried out by trained interviewers who introduced the study to eligible individuals and obtained verbal informed consent.

### Inclusion/exclusion criteria

Respondents were women aged over 50 years who were resident in Wales and gave verbal consent. Women were excluded if they reported having had a personal diagnosis of ovarian cancer and/or had undergone oophorectomy.

### Procedures

Random probability sampling was used to achieve a population-representative sample using electronic telephone directories as the sampling frame. The final two digits of each selected telephone number were replaced with two random numbers, to include numbers that were not publicly available. Households were eligible if one or more person was aged 50 or over and spoke English. Where more than one person was eligible, the Rizzo method was used to randomly select one person to be interviewed, thereby giving an equal chance of selection to all eligible people living in the household [34]. Survey data were collected during May to July 2011 using computer-assisted telephone interviews. At the end of the interview, participants were offered contact details of a local cancer support charity.

### Sample size

Assuming a design effect of 1.2 (adjusting for the impact of the weighting scheme employed), a sub-sample of 1000 women was estimated to provide conservative 95% confidence intervals of +/−3.7%.

### Measures

A survey instrument (ABC-O; Awareness and Beliefs about Cancer-Ovarian) was adapted from the internationally validated Awareness and Beliefs about Cancer measure ABC; [35], and the Cancer Awareness Measure CAM; [36] and its ovarian-specific version [37]. ABC-O questions were tested for comprehensibility using cognitive interviews (n = 10), for test-retest reliability (n = 100), and for content validity using expert ratings (n = 8) of relevance and representativeness. Anticipated time to presentation questions were placed ahead of the symptom recognition question, and the order of all other questions and response options was rotated randomly. Major news stories relating to cancer and cancer awareness campaigns were monitored two weeks prior to and during the survey data collection period. None observed during this period was related to ovarian cancer symptom awareness.

#### Ovarian cancer symptom awareness

Eleven statements about recognition of ovarian cancer symptoms were presented using the question “I’m now going to list some symptoms that may or may not be warning signs for ovarian cancer. For each one, can you tell me whether you think that it could be a warning sign for ovarian cancer?” The list of symptoms included persistent pain in the abdomen, persistent pain in the pelvis, vaginal bleeding after the menopause, persistent bloating, increased abdominal size, feeling full persistently, difficulty eating, passing more urine than usual, a change in bowel habits, extreme tiredness, and back pain (response options were yes, no, don’t know). Items were adapted from the validated ovarian CAM [37] and included less common symptoms (change in bowel habit, fatigue, back pain) to reflect the UK Department of Health’s ‘Key Messages’ on ovarian cancer for health professionals and the public [11,15]. The number of symptoms endorsed was summed (total score range 0–11).

#### Anticipated delay

An open-ended question was used to assess anticipated time to symptomatic presentation: “If you had a symptom that you thought might be a sign of ovarian cancer, please tell me how long it would take you to go to the doctors from the time you first noticed the symptom.” Responses were coded according to a number of predefined categories (e.g., “I would go as soon as I noticed”, “up to one week”, “more than a month”). A dichotomous delay variable (< 3 weeks, > 3 weeks) was created to reflect guidelines regarding frequency and persistence of symptoms such as bloating and pain, and the three week symptom timeline currently used in the UK ovarian cancer awareness campaign [38]. Sensitivity analyses were used to test effects of using different delay thresholds (1 and 2 weeks).

#### Health beliefs

Health beliefs included perceived benefits of early symptomatic presentation, emotional barriers to presentation, practical barriers to presentation, perceived risk, and confidence in symptom detection. *Perceived benefits* included five items (e.g. “If ovarian cancer is diagnosed early, it can be treated more successfully”) rated from 1 (strongly disagree) to 4 (strongly agree) with a total possible score range of 5–20 (Cronbach’s α = 0.71). Four items measured *emotional barriers* (e.g. “I would be too scared”, score range 4–12, α = 0.68). Three items measured *practical barriers* (e.g. “I would be too busy to make time to go to the doctor”, score range 3**–**9, α = 0.60). Response options for the barriers items were 1 = yes, often, 2 = yes, sometimes, and 3 = no (reverse scored). *Perceived risk* was a single item adapted from previous research [39], with response options from 1 (much more likely to get it) to 5 (much less likely to get it) recoded so that a higher score indicated higher perceived risk. *Confidence in symptom detection* was measured by asking respondents “How confident, or not, are you that you would notice a symptom of ovarian cancer?” (1 = not at all confident and 4 = very confident)*.*

#### Cancer worry

The Ovarian Cancer Worry Scale [40] included three items regarding the frequency of worry (“How often do you worry about getting ovarian cancer someday?”), and the impact of worry on mood (“How often, if at all, does your worry about getting ovarian cancer someday affect your mood?”) and functioning (“How often, if at all, does your worry about getting ovarian cancer someday affect your ability to perform your daily activities?”). Items were rated from 1 (not at all) to 5 (almost all the time), with a score range 1–15 (α = 0.69). Scores were log transformed due to non-normal distribution (floor effect).

*Demographic variables* included age, ethnicity, level of education, socioeconomic status (Welsh Index of Material Deprivation score), relationship status, and experience of ovarian cancer diagnosed in family members or friends.

### Statistical analysis

Survey response rate was calculated using the American Association for Public Opinion Research (AAPOR) conventions, because the denominator of eligible people was unknown and therefore response rate could not be calculated in the usual way [41]. The ‘minimum response rate’ was conservatively calculated as the number of complete interviews divided by the number of all possible interviews (the number of interviews among eligible people plus the number of households where eligible people were known to live, but where the interview could not be completed (e.g. refusal, interview broken off) plus the number of all households of unknown eligibility). It represents the response rate assuming that all households that we could not assess for eligibility were eligible (equivalent to AAPOR response rate formula 1). It is likely to underestimate response rates because it is likely that many households were ineligible. We also calculated the ‘estimated response rate’ as the number of completed interviews divided by the estimated number of eligible individuals, based on the proportion of households that were eligible out of those assessed for eligibility (equivalent to AAPOR response rate formula 3).

Associations between demographic variables and ovarian symptom awareness were examined using appropriate univariate analyses. Preliminary associations between anticipated delay and demographic variables, symptom awareness, health beliefs and cancer worry were tested using chi square or independent t-tests, with variables significant at p ≤ 0.01 subsequently entered into a logistic regression model. Results are presented for both unadjusted data and data adjusted for sample non-representativeness in age, region, relationship status and education. Sensitivity analyses were undertaken at each stage to test for effects of under-representation of certain demographic groups.

## Results

### Sample characteristics

The overall study response rate was 2298 eligible men and women completing the larger ABC survey in Wales (Table 1). The minimum response rate was 10.5% because the number of households for which we did not know eligibility was high, due to the use of random digit dialling. The estimated response rate was 46.8%.

It was not possible to determine the number of eligible women: of the 2298 survey respondents, 1385 respondents were female. A total 315 women (26%) were excluded due to a personal medical history of ovarian cancer (n = 19) or oophorectomy (n = 296). The final sample was 1043.

**Table 1:** Overall response rate

	**N**
Total number of households with connected telephone numbers approached	26,262
Number of households of unknown eligibility*	18,210
Number of households of known eligibility	8,052
Number of households in which the individual declined to take part either during or after assessment of eligibility	1,294
Number of ineligible households*	4,283
Number of eligible households*	3,769
Proportion of households eligible among those assessed for eligibility (%)	46.8
Completed interviews	2,298
**Minimum response rate (%)**^**†**^	**10.5**
Estimated response rate (%)**	**46.8**

*A household was eligible if one or more people aged 50+ lived in the household.

^**†**^The minimum response rate represents the response rate assuming all households that we could not assess for eligibility were eligible, in other words the lowest possible response rate. It is calculated as the number of completed interviews divided by the number of all possible interviews, i.e. the number of interviews among eligible people plus the number of incomplete interviews among eligible people (refusals, break-offs and non-contacts) plus the number of all households of unknown eligibility (equivalent to the American Association for Public Opinion Research response rate formula 1).

**The estimated response rate represents the response rate after adjusting the size of the denominator for the likely proportion of households that were eligible. It is calculated by assuming that the proportion eligible among households of unknown eligibility is the same as the proportion of those tested for eligibility who were eligible (equivalent to American Association for Public Opinion Research response rate formula 3).

As shown in Table 2, most respondents were aged over 60 years and of white ethnicity. Half the sample was not married or cohabiting, more than half had been educated up to 16 years only, and almost a quarter had experience of ovarian cancer. Most women anticipated presenting within one week of noticing a potential ovarian symptom.

**Table 2:** Sample characteristics (N = 1043)

**Variable**	**Descriptive statistic**	
***Age, years *** **n (%)**		
50-59	348	(33.4%)
60-69	387	(37.0%)
70+	300	(28.8%)
Missing	8	(0.8%)
***Ethnic background *** **n (%)**		
White ethnicity	1031	(98.8%)
Other ethnicity	11	(1.1%)
Missing	1	(0.1%)
***Relationship status *** **n (%)**		
Married or cohabiting	515	(49.4%)
Not married or cohabiting	525	(50.3%)
Missing	3	(0.3%)
***Education *** **n (%)**		
Up to 16 years	570	(54.7%)
Secondary	254	(24.4%)
Degree and above	197	(18.9%)
Missing	22	(2.0%)
***Socioeconomic status***		
First quartile (most deprived)	178	(17.1%)
Second quartile	246	(23.6%)
Third quartile	229	(22.0%)
Fourth quartile (least deprived)	253	(24.3%)
Missing	137	(13.0%)
***Experience of ovarian cancer *** **n (%)**		
Experience of ovarian cancer	238	(22.8%)
No experience of ovarian cancer	800	(76.7%)
Missing	5	(0.5%)
***Anticipated delay *** **n (%)**		
I would go as soon as I noticed	507	(48.6%)
Up to one week	239	(22.9%)
Over 1 up to 2 weeks	101	(9.7%)
Over 2 up to 3 weeks	51	(4.9%)
Over 3 up to 4 weeks	57	(5.5%)
More than a month	43	(4.1%)
I would not contact my doctor	8	(0.8%)
I would go to a nurse instead of my doctor^1^	3	(0.3%)
Missing	34	(3.3%)

^1^Coded as missing.

### Ovarian symptom awareness levels

**Figure 1:**
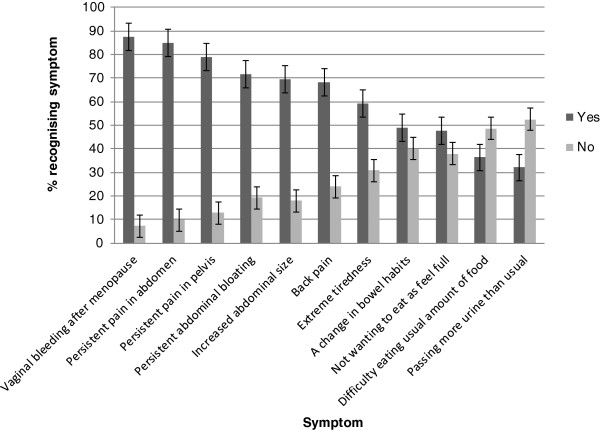
Recognition of individual ovarian cancer symptoms.

As shown in Figure 1, the most well recognised symptoms were post-menopausal vaginal bleeding (87.4%), abdominal pain (85.0%), and pelvic pain (79.0%). More than half the sample was able to recognise abdominal bloating (71.7%), increased abdominal size (69.4%), back pain (68.3%) and tiredness (59.1%). The least recognised symptoms included a change in bowel habits (49.0%), feeling full quickly (47.7%), difficulty eating (36.3%), and a change in bladder habits (32.0%). The mean symptom recognition score was 6.85 (SD 2.73, range 0–11).

### Risk factors for low ovarian symptom awareness

There was a significant effect of age on symptom awareness (p ≤ 0.001), indicating that awareness was significantly lower in participants aged 70+ compared to those aged 50–59 and 60–69 (Table 3). Participants who were not married/cohabiting (p ≤ 0.001), educated up to 16 years (p ≤ 0.01), and without experience of ovarian cancer (p ≤ 0.01) reported lower awareness. There was a marginal effect of lower socioeconomic status (p ≤ 0.05). The association between awareness and anticipated delay was not significant. A similar pattern of results was observed after adjusting for sample non-representativeness; however, the relationship between lower awareness and anticipated delay reached statistical significance (p ≤ 0.01).

**Table 3:** Risk factors for low ovarian symptom awareness

	**Mean (sd) number of ovarian symptoms recognised out of 11**			
	**Unadjusted**	**Statistic**	**Adjusted**^**1**^	**Statistic**
***Age groups***				
50-59 years	7.06 (2.61)	*F* (_2, 1032_) = 10.18, p = 0.000***	7.23 (2.62)	*F* (_2, 1018_) = 16.93, p = 0.000***
60-69 years	7.13 (2.61)		7.00 (2.65)	
70+ years	6.27 (2.92)		6.08 (2.94)	
***Ethnic background***				
White ethnicity	6.85 (2.74)	^	6.73 (2.80)	^
Other ethnicity	7.18 (2.36)		6.86 (2.41)	
***Relationship status***				
Married or cohabiting	7.16 (2.56)	*t* (_1038_) = 3.65, p = 0.000***	7.08 (2.61)	*t* (_1025_) = 4.41, p = 0.000***
Not married or cohabiting	6.55 (2.86)		6.30 (2.97)	
***Education***				
Up to 16 years	6.58 (2.81)	*F* (_2, 1018_) = 6.34, p = 0.002**	6.49 (2.85)	*F* (_2, 1005_) = 8.23, p = 0.000***
Secondary	7.16 (2.58)		7.08 (2.60)	
Degree and above	7.21 (2.57)		7.37 (2.56)	
***Socioeconomic status***				
First quartile (most deprived)	6.39 (2.75)	*F* (_3,902_) = 2.82, p = 0.04*	6.29 (2.74)	*F* (_3,886_) = 2.82, p = 0.03*
Second quartile	7.07 (2.68)		7.02 (2.72)	
Third quartile	7.10 (2.74)		6.85 (2.84)	
Fourth quartile (least deprived)	6.87 (2.65)		6.56 (2.86)	
***Experience of ovarian cancer***				
Experience of ovarian cancer	7.23 (2.52)	*t* (_1036_) = 2.51, p = 0.01**	7.32 (2.43)	*t* (_1021_) = 3.84, p = 0.000***
No experience of ovarian cancer	6.75 (2.78)		6.59 (2.86)	
***Anticipated delay***				
Up to three weeks	6.99 (2.67)	*t* (_1004_) = 1.57, p = 0.12	6.90 (2.75)	*t* (_993_) = 0.27, p = 0.006**
More than three weeks	6.56 (2.83)		6.11 (2.83)	

*p ≤ .05, **p ≤ .01, *** *p* ≤ .001, ^ sample size not large enough in some cells to conduct statistical tests.

*F* = ANOVA, *t* = independent t-test, *r* = Pearson’s correlation.

^1^Adjusted for sample non-representativeness in age, education, region, and relationship status.

### Risk factors for anticipated delay

Table 4 displays preliminary associations between independent variables and anticipated delay. Women in the 50–59 age group (p ≤ 0.01) and those educated to degree level (p ≤ 0.001) were significantly more likely to anticipate waiting at least three weeks. Anticipated delay was significantly associated with reporting more emotional barriers (p ≤ 0.001), more practical barriers (p ≤ 0.001), and lower confidence in symptom detection (p ≤ 0.001). There were no significant effects of relationship status, socioeconomic status, ovarian cancer experience, cancer worry, perceived risk, or perceived benefits of early presentation. Analyses were repeated after weighting for non-representativeness, with little observed difference other than cancer worry reaching marginal significance (p ≤ 0.05).

**Table 4:** Preliminary analysis of risk factors for anticipated delay

	**<3 weeks**	**>3 weeks**		**<3 weeks**	**>3 weeks**	
	**(n = 898)**	**(n = 108)**		**(n = 894)**	**(n = 100)**	
	**Unadjusted**		**Statistic**	**Adjusted**^**1**^		**Statistic**
***Age groups *** **n (%)**						
50-59 years	287 (84)	53 (16)	χ^*2*^_(2)_ = 13.36, p = 0.01**	299 (86)	48 (14)	χ^2^_(2)_ = 9.20, p = 0.01**
60-69 years	337 (90)	36 (10)		265 (90)	28 (10)	
70+ years	266 (93)	19 (7)		322 (93)	24 (7)	
***Ethnic background *** **n (%)**						
White ethnicity	888 (89)	106 (11)	^	884 (90)	97 (10)	^
Other ethnicity	9 (82)	2 (18)		9 (75)	3 (25)	
***Relationship status *** **n (%)**						
Married or cohabiting	450 (90)	49 (10)	χ^*2*^_(1)_ = 0.74, p = 0.39	498 (91)	52 (10)	χ^*2*^_(1)_ = 0.39, p = 0.53
Not married or cohabiting	445 (88)	59 (12)		394 (89)	48 (11)	
***Education *** **n (%)**						
Up to 16 years	502 (92)	46 (8)	χ^*2*^_(2)_ = 18.59, p = 0.000***	613 (92)	52 (8)	χ^2^_(2)_ = 25.75, p = 0.000***
Secondary	223 (90)	24 (10)		135 (91)	14 (9)	
Degree and above	152 (80)	37 (20)		125 (79)	34 (21)	
***Socioeconomic status***						
First quartile (most deprived)	156 (91)	15 (9)	χ^*2*^_(3)_ = 3.75, p = 0.29	176 (92)	16 (8)	χ^*2*^_(3)_ = 6.73, p = 0.08
Second quartile	215 (91)	21 (9)		219 (93)	16 (7)	
Third quartile	207 (92)	18 (8)		198 (90)	22 (10)	
Fourth quartile (least deprived)	211 (87)	31 (13)		181 (86)	29 (14)	
***Experience of ovarian cancer *** **n (%)**						
Experience of ovarian cancer	201 (87)	29 (13)	χ^*2*^_(1)_ = 1.02, p = 0.31	191 (88)	26 (12)	χ^*2*^_(1)_ = 1.05, p = 0.31
No experience of ovarian cancer	694 (90)	77 (10)		699 (91)	72 (9)	
***Ovarian cancer worry *** **m (sd)**	1.31 (0.32)	1.28 (0.27)	*t*_(1002)_ = 0.94, p = 0.35	1.31 (0.33)	1.24 (0.24)	*t*_(991)_ = 1.99, p = 0.05*
***Health beliefs *** **m (sd)**						
Perceived susceptibility	2.43 (0.95)	2.29 (0.97)	*t*_(913)_ = 1.43, p = 0.15	2.38 (0.98)	2.23 (0.93)	*t*_(896)_ = 1.48, p = 0.14
Perceived benefits	17.29 (2.27)	17.11 (2.42)	*t*_(846)_ = 0.74, p = 0.46	17.24 (2.33)	16.96 (2.65)	*t*_(808)_ = 1.07, p = 0.29
Perceived emotional barriers	4.67 (1.25)	5.43 (1.94)	*t*_(989)_ = −3.96, p = 0.000***	4.72 (1.30)	5.67 (2.11)	*t*_(976)_ = −4.32, p = 0.000***
Perceived practical barriers	3.39 (0.86)	4.40 (1.61)	*t*_(1000)_ = −6.38, p = 0.000***	3.42 (0.92)	4.50 (1.67)	*t*_(989)_ = −6.58, p = 0.000***
Confidence in symptom detection	2.44 (0.91)	1.86 (0.76)	*t*_(980)_ = 7.30, p = 0.000***	2.46 (0.93)	1.77 (0.70)	*t*_(959)_ = 8.89, p = 0.000***

*p ≤ .05, **p ≤ .01, ***p ≤ .001, ^ sample size not large enough in some cells to conduct statistical tests.

χ^*2*^ = chi square test, *t* = independent t test.

^1^ Adjusted for sample non-representativeness in age, education, region, and relationship status.

Statistically significant variables were modelled to determine their effects on anticipated delay. As shown in Table 5, the full model was statistically significant (χ^*2*^ (6) = 107.61, p ≤ 0.001) and explained 11% – 22% of the variance in anticipated delay, correctly classifying 89% of cases. The strongest determinant of anticipated delay was being educated to degree level (OR = 2.64, p ≤ 0.001). Women who reported more practical barriers (OR = 1.60, p ≤ 0.001), less confidence in symptom detection (OR = 0.56, p ≤ 0.001), and more emotional barriers (OR = 1.21, p ≤ 0.01) were more likely to anticipate waiting at least three weeks. Neither age nor ovarian symptom awareness showed a statistically significant association with anticipated delay.

**Table 5:** Logistic regression predicting the likelihood of anticipated delay (< / > 3 weeks)

	**Unadjusted**					**Adjusted**^**1**^				
				**Lower**	**Upper**				**Lower**	**Upper**
**Variables**	**B (SE)**	**p**	**OR**	**95% CI**	**95% CI**	**B (SE)**	**p**	**OR**	**95% CI**	**95% CI**
Age (0 = 60+, 1 = 50-59)	0.31 (0.24)	0.20	1.36	0.86	2.15	0.07 (0.26)	0.80	1.07	0.64	1.79
Education (0 = up to degree, 1 = degree+)	0.97 (0.25)	≤.001	2.64	1.61	4.33	1.34 (0.28)	≤.001	3.83	2.21	6.64
Ovarian cancer symptom awareness (0–11)	−0.02 (0.04)	0.74	0.99	0.91	1.07	−0.07 (0.04)	0.11	0.93	0.85	1.02
Practical barriers (3–9)	0.47 (0.09)	≤.001	1.60	1.34	1.91	0.43 (0.09)	≤.001	1.54	1.29	1.83
Emotional barriers (4–12)	0.19 (0.07)	≤.01	1.21	1.06	1.40	0.23 (0.07)	≤.001	1.26	1.10	1.46
Confidence (1–4)	−0.59 (0.14)	≤.001	0.56	0.42	0.73	−0.70 (0.15)	≤.001	0.50	0.37	0.68

OR = odds ratio, CI = confidence interval.

^1^Adjusted for sample non-representativeness in age, education, region, and relationship status.

Repeating the regression analysis on the weighted data had little effect. The full model was statistically significant (χ^*2*^ (6) = 124.31, p ≤ 0.001) and explained 13% – 26% of the variance in anticipated presentation, correctly classifying 90% of cases. The pattern of significant determinants remained the same. In addition, sensitivity analyses confirmed the use of a three week threshold to reflect anticipated delay.

## Discussion

Once known as “the silent killer”, ovarian cancer is increasingly recognised as having identifiable early symptoms. However, until an effective method of ovarian screening is found, women’s prompt help-seeking when they have a potential symptom remains an important avenue to early detection. This population-based survey found that many symptoms of ovarian cancer were not well recognised by women in the general population. Ovarian symptoms associated with pain, bloating and abnormal bleeding were better recognised than those associated with eating difficulties and changes in bowel and bladder habits. Accurate recognition may be especially difficult due to the vague and non-specific nature of some ovarian cancer symptoms, which may be mistaken for benign conditions [10]. In addition, awareness of ovarian cancer symptoms was not strongly related to anticipated delay. The marginal association that was observed between symptom awareness and anticipated delay in the present context may reflect the ambiguity of many early symptoms of ovarian cancer. Improving public awareness of potential early symptoms could contribute to earlier detection of ovarian cancer, but this requires clinical evidence and consensus regarding what symptom information and guidance should be provided to the public.

Factors associated with poorer recognition of ovarian symptoms included older age, being single, lower educational level, and lack of personal experience of the condition. Similarly, Grunfeld et al. [23] found that women aged over 65 had low knowledge of breast symptoms and lower perceived risk of breast cancer compared to younger women. The risk of developing ovarian cancer increases with age, yet poor knowledge and absence of concern about ovarian cancer may mean that symptoms experienced by older women are attributed to other causes such as the menopause or ageing process, rather than recognised as a potential threat to health. This may especially be the case for older women who lack a spouse or confidante with whom to disclose symptoms [22,24]. Educational initiatives could therefore target public understanding of the age/risk association for ovarian cancer.

In contrast to the findings for awareness, the strongest risk factor for anticipated delay was higher education. Other important determinants of delay included lack of confidence in detecting ovarian cancer symptoms, practical barriers such as being too busy and not wanting to waste the doctor’s time, and emotional factors such as fear and embarrassment. These finding highlight the importance of a range of psychological, social and behavioural barriers that may impede the decision to act on a suspected symptom. Beliefs about self-care, downplaying potentially serious symptoms, and waiting to see if symptoms resolve by themselves are important barriers to prompt presentation [26]. Similarly, Scott et al. [42] found that procrastinating about help-seeking for oral cancer symptoms was strongly linked to competing priorities and concerns about the consultation, such as fear of consequences and not wanting to bother the doctor. The finding regarding educational level contrasts with reported associations between lower education and delayed presentation for breast and colon cancer [25], but mirrors the findings of Low et al. [43]. Further qualitative research may help to understand the links between higher education and perceived barriers to presentation with ovarian symptoms, in particular the perception of time-wasting. Improving women’s confidence may be necessary to bridge the gap between ovarian cancer symptom awareness and earlier presentation, for example by providing an explicit action plan that describes how and when to act on potential ovarian symptoms [20,42,44], including timely follow-up investigations and onward referrals [45,46] based on clinical consensus regarding ovarian symptom duration and threshold.

Health beliefs relating to perceived benefits of early presentation were not statistically associated with symptom awareness or anticipated delay. Overall, women perceived their risk of ovarian cancer to be average/low and held positive beliefs, reflecting the overall lack of concern about ovarian cancer within a population sample. While a moderate amount of concern or worry may have a beneficial role in prompting health behaviour [47], it was not possible to test this due to floor effects (i.e. very low worry scores). Comparison with women at increased risk due to a family history of ovarian cancer would help to illuminate the role of emotions in appraising and acting on ovarian symptoms.

The hypothetical nature of the health threat and cross-sectional design are potential limitations of the current study. Since intentions do not always translate into actual help-seeking behaviour [48], the relationship between cancer symptom awareness and actual presentation would ideally be tested in large-scale prospective studies [49,50]. The limited association that was found between ovarian symptom awareness and anticipated delay contrasts with Robb et al. [3], who found a modest significant association between higher recognition of general cancer symptoms and shorter anticipated presentation. This may reflect the use of an aggregated ovarian symptom recognition measure in the current study, which may have diluted any effects of specific symptom recognition [51]. With a larger sample, it may be possible to test whether recognition of specific ovarian symptoms such as pain, bloating and abnormal bleeding reduces the risk of delayed presentation [22,27].

## Conclusions

Many ovarian symptoms were not well recognised by women in the general population. Risk factors for delayed presentation included higher education, perceived barriers, and low confidence in detecting ovarian cancer symptoms. Further clinical research is needed to develop evidence-informed ovarian cancer early diagnosis strategies and action plans, and to inform the nuances of the ovarian cancer symptom message. Interventions could attempt to overcome the barriers to timely symptomatic presentation, for example by improving public understanding of the age/risk association for ovarian cancer and improving women’s confidence in their personal abilities to recognise and act upon ovarian symptoms.

## Competing interests

The authors declare that they have no competing interests.

## Authors’ contributions

KB conceived and designed the study, participated in data acquisition, supervised data analysis and interpretation, and drafted the manuscript. SS carried out the statistical analysis, and assisted with data interpretation and manuscript preparation. AES, LF and CR made a substantial contribution to study conception and design, participated in data acquisition, and contributed to data interpretation and writing. IJR, JS, CW, RN and JH participated in drafting the manuscript and revising it critically for important intellectual content. All authors read and approved the final manuscript.

## Pre-publication history

The pre-publication history for this paper can be accessed here:

http://www.biomedcentral.com/1471-2407/14/171/prepub

## References

[B1] Cancer research UK ovarian cancer UK statistics [http://publications.cancerresearchuk.org/downloads/Product/cs_pdf_ovarian_march_2011.pdf]

[B2] Maringe C Walters S Butler J Coleman MP Hacker N Hanna L Mosgaard BJ Nordin A Rosen B Engholm G Gjerstorff ML Hatcher J Johannesen TB McGahan CE Meechan D Middleton R Tracey E Turner D Richards MA Rachet B ICBP Module 1 Working Group Stage at diagnosis and ovarian cancer survival: evidence from the international cancer benchmarking partnership Gynecol Oncol 2012 127 75 82 10.1016/j.ygyno.2012.06.033 22750127

[B3] Richards MA Westcombe AM Love SB Littlejohns P Ramirez AJ Influence of delay on survival in patients with breast cancer: a systematic review Lancet 1999 353 1119 1126 10.1016/S0140-6736(99)02143-1 10209974

[B4] Wardle J Waller J Brunswick N Jarvis MJ Awareness of risk factors for cancer among British adults Public Health 2001 115 173 174 10.1038/sj.ph.1900752 11429711

[B5] Robb K Stubbings S Ramirez A Macleod U Austoker J Waller J Hiom S Wardle J Public awareness of cancer in Britain: a population-based survey of adults Brit J Cancer 2009 101 18 123 10.1038/sj.bjc.6605386 PMC2790705 19956158

[B6] Coleman MP Forman D Bryant H Butler J Rachet B Maringe C Nur U Tracey E Coory M Hatcher J McGahan CE Turner D Marrett L Gjerstorff ML Johannesen TB Adolfsson J Lambe M Lawrence G Meechan D Morris EJ Middleton R Steward J Richards MA ICBP Module 1 Working Group Cancer survival in Australia, Canada, Denmark, Norway, Sweden, and the UK, 1995—2007 (the international cancer benchmarking partnership): an analysis of population-based cancer registry data Lancet 2011 377 127 138 10.1016/S0140-6736(10)62231-3 21183212 PMC3018568

[B7] Bankhead C Collins C Stokes-Lampard H Rose P Wilson S Clements A Mant D Kehoe S Austoker J Identifying symptoms of ovarian cancer: a qualitative and quantitative study Brit J Obs Gyn 2008 115 1008 1014 10.1111/j.1471-0528.2008.01772.x PMC2607526 18651882

[B8] Hamilton W Peters TJ Bankhead C Sharp D Risk of ovarian cancer in women with symptoms in primary care: population based case–control study BMJ 2009 339 b2998 doi:10.1136/bmj.b2998 10.1136/bmj.b2998 19706933 PMC2731836

[B9] Goff BA Mandel L Muntz HG Melancon CH Ovarian carcinoma diagnosis: results of a national ovarian cancer survey Cancer 2000 89 2068 2075 10.1002/1097-0142(20001115)89:10<2068::AID-CNCR6>3.0.CO;2-Z 11066047

[B10] Smith EM Anderson B The effects of symptoms and delay in seeking diagnosis on stage of disease at diagnosis among women with cancers of the ovary Cancer 1985 56 2727 2732 10.1002/1097-0142(19851201)56:11<2727::AID-CNCR2820561138>3.0.CO;2-8 4052947

[B11] Department of Health key messages for ovarian cancer for health professionals [http://webarchive.nationalarchives.gov.uk/20130107105354/http://www.dh.gov.uk/en/Publicationsandstatistics/Publications/PublicationsPolicyAndGuidance/DH_110534]

[B12] National Institute for Health and Clinical Excellence (NICE) guidance Ovarian Cancer: The Recognition and Initial Management of Ovarian Cancer 2011 Cardiff: National Collaborating Centre for Cancer

[B13] Hippisley-Cox J Coupland C Vinogradova Y Robson J May M Brindle P Derivation and validation of QRISK, a new cardiovascular disease risk score for the United Kingdom: prospective open cohort study BMJ 2007 335 136 10.1136/bmj.39261.471806.55 17615182 PMC1925200

[B14] Cancer Facts & Figures 2011 Atlanta: American Cancer Society [http://www.cancer.org/acs/groups/content/@epidemiologysurveilance/documents/document/acspc-029771.pdf]

[B15] Department of Health key messages for ovarian cancer for members of the public [http://www.eveappeal.org.uk/media/42340/km_ovarian.pdf]

[B16] Richards MA The national awareness and early detection initiative: size of the prize for earlier diagnosis of cancer in England Brit J Cancer 2009 101 S125 S129 19956156 10.1038/sj.bjc.6605402 PMC2790715

[B17] National Audit of Cancer Diagnosis in Primary Care 2011 London: Royal College of General Practitioners [http://www.rcgp.org.uk/news/2011/november/~/media/Files/News/National_Audit_of_Cancer_Diagnosis_in_Primary-Care.ashx]

[B18] Goff BA Mandel LS Drescher CW Urban N Gough S Schurman KM Patras J Mahony BS Andersen MR Development of an ovarian cancer symptom index: possibilities for earlier detection Cancer 2007 109 221 227 10.1002/cncr.22371 17154394

[B19] Andersen MR Goff BA Lowe KA Scholler N Bergan L Dresher CW Paley P Urban N Combining a symptoms index with CA 125 to improve detection of ovarian cancer Cancer 2008 113 484 489 10.1002/cncr.23577 18615684 PMC2734274

[B20] Andersen MR Goff BA Lowe KA Scholler N Bergan L Dresher CW Paley P Urban N Use of a Symptom Index, CA125 and HE4 to predict ovarian cancer Gynecol Oncol 2010 116 378 383 10.1016/j.ygyno.2009.10.087 19945742 PMC2822097

[B21] Menon U Gentry-Maharaj A Hallett R Ryan A Burnell M Sharma A Lewis S Davies S Philpott S Lopes A Godfrey K Oram D Herod J Williamson K Seif MW Scott I Mould T Woolas R Murdoch J Dobbs S Amso NN Leeson S Cruickshank D McGuire A Campbell S Fallowfield L Singh N Dawnay A Skates SJ Parmar M Jacobs I Sensitivity and specificity of multimodal and ultrasound screening for ovarian cancer, and stage distribution of detected cancers: results of the prevalence screen of the UK Collaborative Trial of Ovarian Cancer Screening (UKCTOCS) Lancet Oncol 2009 10 327 340 10.1016/S1470-2045(09)70026-9 19282241

[B22] Ramirez AJ Westcombe AM Burgess CC Sutton S Littlejohns P Richards MA Factors predicting delayed presentation of symptomatic breast cancer: a systematic review Lancet 1999 353 1127 1131 10.1016/S0140-6736(99)02142-X 10209975

[B23] Grunfeld E Ramirez A Hunter M Richards M Women’s knowledge and beliefs regarding breast cancer Brit J Cancer 2002 86 1373 1378 10.1038/sj.bjc.6600260 11986766 PMC2375381

[B24] Burgess CC Ramirez A Richards M Love S Who and what influences delayed presentation in breast cancer? Brit J Cancer 1998 77 1343 1348 10.1038/bjc.1998.224 9579844 PMC2150175

[B25] Macleod U Mitchell ED Burgess C Macdonald S Ramirez AJ Risk factors for delayed presentation and referral of symptomatic cancer: evidence for common cancers Brit J Cancer 2009 101 92 101 10.1038/sj.bjc.6605398 PMC2790698 19956172

[B26] Smith LK Pope C Botha JL Patients’ help-seeking experiences and delay in cancer presentation: a qualitative synthesis Lancet 2005 366 825 831 10.1016/S0140-6736(05)67030-4 16139657

[B27] Brouha XD Tromp DM Hordijk GJ Winnubst JA de Leeuw JR Oral and pharyngeal cancer: analysis of patient delay at different tumor stages Head Neck 2005 27 939 945 10.1002/hed.20270 16206281

[B28] Cancer in Wales 1995–2009: a comprehensive report 2011 Welsh Cancer Intelligence and Surveillance Unit [http://www.wales.nhs.uk/sites3/Documents/242/8%20Gynaecological%20Cancers.pdf]

[B29] Becker MH The health belief model and sick role behavior Health Educ Monogr 1974 2 409 419

[B30] Bandura A Self-efficacy: The Exercise of Control 1997 New York: Freeman

[B31] Rosenstock IM Strecher VJ Becker HM Social learning theory and the health belief model Health Educ Q 1988 15 175 183 10.1177/109019818801500203 3378902

[B32] Hay JL McCaul KD Magnan RE Does worry about breast cancer predict screening behaviors? A meta-analysis of the prospective evidence Prev Med 2006 42 401 408 10.1016/j.ypmed.2006.03.002 16626796

[B33] Forbes LJL Simon AE Warburton F Boniface D Brain KE Dessaix A Donnelly C Haynes K Hvidberg L Lagerlund M Lockwood G Tishelman C Vedsted P Vigmostad MN Ramirez AJ Wardle J International Cancer Benchmarking Partnership Module 2 Working Group, International Cancer Benchmarking Partnership Programme Board, International Cancer Benchmarking Partnership Module 2 Academic Reference Group Differences in cancer awareness and beliefs between Australia, Canada, Denmark, Norway, Sweden and the UK (the international cancer benchmarking partnership): do they contribute to differences in cancer survival? Brit J Cancer 2013 108 292 300 10.1038/bjc.2012.542 23370208 PMC3566814

[B34] Rizzo L Brick JM Park I A minimally intrusive method for sampling persons in random digit dial surveys Public Opin Q 2004 68 267 274 10.1093/poq/nfh014

[B35] Simon AE Forbes LJL Boniface D Warburton F Brain KE Dessaix A Donnelly M Hayne K Hvidberg L Lagerlund M Peterman L Tishelman C Vedsted P Vigmostad MN Wardle J Ramirez AJ ICBP Module 2 Working Group, ICBP Programme Board and Academic Reference Group An international measure of awareness and beliefs about cancer: development and testing of the ABC BMJ Open 2012 2 e001758 doi:10.1136/bmjopen-2012-001758 23253874 10.1136/bmjopen-2012-001758 PMC3547316

[B36] Stubbings S Robb K Waller J Ramirez A Austoker J Macleod U Hiom S Wardle J Development of a measurement tool to assess public awareness of cancer Brit J Cancer 2009 101 s13 s17 19956157 10.1038/sj.bjc.6605385 PMC2790699

[B37] Simon AE Wardle J Grimmett C Power E Corker E Menon U Matheson L Waller J Ovarian and cervical cancer awareness: development of two validated measurement tools J Fam Plann Reprod Health Care 2012 38 167 174 10.1136/jfprhc-2011-100118 21933805 PMC3970720

[B38] Cancer research UK “Be clear on cancer” campaign [http://www.cancerresearchuk.org/cancer-info/spotcancerearly/naedi/beclearoncancer/ovarian/]

[B39] Tyndel S Henderson B Austoker J Brain K Bankhead C Clements A Watson E What is the psychological impact of mammographic screening on younger women with a family history of breast cancer? Findings from a prospective cohort study by the PIMMS management group J Clin Oncol 2007 25 3823 3830 10.1200/JCO.2007.11.0437 17761970

[B40] Andersen MR Drescher CW Zheng Y Bowen DJ Wilson S Young A McIntosh M Mahony BS Lowe KA Urban N Changes in cancer worry associated with participation in ovarian cancer screening Psycho-Oncol 2007 16 814 820 10.1002/pon.1151 17225260

[B41] AAPOR Standard Definitions: Final Dispositions of Case Codes and Outcome Rates for Surveys 2011 7 The American Association of Public Opinion Research http://www.aapor.org/AM/Template.cfm?Section=Standard_Definitions2&Template=/CM/ContentDisplay.cfm&ContentID=3156

[B42] Scott SE Grunfeld EA Auyeung V Barriers and triggers to seeking help for potentially malignant oral symptoms: implications for interventions Amer Assoc Publ Health Dentistry 2009 69 34 40 10.1111/j.1752-7325.2008.00095.x 18662249

[B43] Low EL Waller J Menon U Jones A Reid F Simon AE Ovarian cancer symptom awareness and anticipated time to help-seeking for symptoms among UK women J Fam Plann Reprod Health Care 2013 39 163 171 10.1136/jfprhc-2012-100473 23709609

[B44] Gollwitzer PM Goal achievement: the role of intentions Eur Rev Soc Psychol 1993 4 141 185 10.1080/14792779343000059

[B45] Neal RD Do diagnostic delays in cancer matter? Brit J Cancer 2009 101 S9 S12 19956171 10.1038/sj.bjc.6605384 PMC2790696

[B46] Evans J Ziebland S McPherson A Minimizing delays in ovarian cancer diagnosis: an expansion of Anderson’s model of ‘total patient delay’ Fam Prac 2006 24 48 55 10.1093/fampra/cml063 17158183

[B47] Andersen MR Smith R Meischke H Bowen D Urban N Breast cancer worry and mammography use by women with and without a family history in a population-based sample Cancer Epid, Bio Prev 2003 12 314 320 12692105

[B48] Sheeran P Intention-behaviour relations: a conceptual and empirical review Eur Rev Soc Psychol 2002 12 1 36 10.1080/14792772143000003

[B49] Andersen RS Vedsted P Olesen F Bro F Søndergaard J Patient delay in cancer studies: a discussion of methods and measures BMC Health Serv Res 2009 9 189 10.1186/1472-6963-9-189 19840368 PMC2770466

[B50] Simon AE Waller J Robb K Wardle J Patent delay in presentation of possible cancer symptoms: the contribution of knowledge and attitudes in a population sample from the United Kingdom Cancer Epid, Bio Prev 2010 19 2272 2277 10.1158/1055-9965.EPI-10-0219 PMC2938472 20660602

[B51] Quaife S Forbes L Ramirez A Brain KE Donnelly C Simon AE Wardle J Recognition of cancer warning signs and anticipated delay in help-seeking in a population sample of adults in the UK Brit J Cancer 2013 10.1038/bjc.2013.684 10.1038/bjc.2013.684 PMC3887291 24178761

